# Prevalence Studies of Dementia in Mainland China, Hong Kong and Taiwan: A Systematic Review and Meta-Analysis

**DOI:** 10.1371/journal.pone.0066252

**Published:** 2013-06-11

**Authors:** Yu-Tzu Wu, Hsin-yi Lee, Samuel Norton, Chuanfeng Chen, Hongxia Chen, Chenglin He, Jane Fleming, Fiona E. Matthews, Carol Brayne

**Affiliations:** 1 Department of Public Health and Primary Care, Institute of Public Health, University of Cambridge, Cambridge, United Kingdom; 2 Department of Health, Behavior and Society, Johns Hopkins Bloomberg School of Public Health, Baltimore, Maryland, United States of America; 3 Psychology Department, Institute of Psychiatry, King’s College London, London, United Kingdom; 4 Institute of Psychological Science and Health Education, College of Teacher Education, Huzhou Teachers College, Huzhou city, Zhejiang province, People’s Republic of China; 5 Department of Psychology, College of Teacher Education, Ningbo University, Zhejiang Province, People’s Republic of China; 6 Medical Research Council Biostatistics Unit, Institute of Public Health, University of Cambridge, United Kingdom; Banner Alzheimer's Institute, United States of America

## Abstract

**Background:**

Many studies have considered the prevalence of dementia in mainland China, Hong Kong and Taiwan. However, area level estimates have not been produced. This study examines area differences across mainland China, Hong Kong and Taiwan adjusting for the effect of methodological factors with the aim of producing estimates of the numbers of people with dementia in these areas.

**Method and Findings:**

A search of Chinese and English databases identified 76 dementia prevalence studies based on samples drawn from mainland China, Hong Kong and Taiwan between 1980 and 2012. A pattern of significantly decreasing prevalence was observed from northern, central, southern areas of mainland China, Hong Kong and Taiwan. Area variations in dementia prevalence were not explained by differences in methodological factors (diagnostic criteria, age range, study sample size and sampling method), socioeconomic level or life expectancy between areas. The results of meta-analysis were applied to current population data to provide best estimate. Based on the DSM-IV diagnostic criteria, the total number of people aged 60 and over with dementia in mainland China, Hong Kong and Taiwan is 8.4 million (4.6%, 95% CI: 3.4, 5.8) and in northern, central and southern areas are 3.8 (5.1%, 95% CI: 4.1, 6.1), 3.2 (4.4%, 95% CI: 3.2, 5.6) and 1.2 (3.9%, 95% CI: 2.3, 5.4) million respectively. These estimates were mainly based on the studies existing in highly developed areas and potentially affected by incomplete and insufficient data.

**Conclusions:**

The findings of this review provide a robust estimate of area differences in dementia prevalence. Application of the estimated prevalence to population data reveals the number of people with dementia is expected to double every 20 years, areas in mainland China will be facing the greatest dementia challenge.

## Introduction

As the country with the largest population in the world, China will face considerable challenges in adapting to its ageing population and including rising numbers of people with dementia [1], [2]. In the immediate and mid term future, investigating the nationwide prevalence of dementia and the distributions in different areas are both important in providing information for appropriate policy and care plans. Some large scale exercises which synthesise Chinese data on dementia do exist but none of them consider the influence of methodological factors on the results [2]–[5]. An international Lancet report (Delphi study) seven years ago concluded the consensus prevalence of dementia in China and the developing western Pacific as 4.0% in those aged over 60 years [3]. A more recent meta-analysis carried out for the World Health Organisation global study of dementia produced a crude estimate of 3.2% in the population aged 60 and over and 4.98% after age and sex standardisation to the population structure of Western Europe [2].

Both these international reviews produced similar estimates of dementia prevalence for China. However, heterogeneity between individual prevalence studies is substantial. Dong et al. (2007) reviewed 25 studies in mainland China from 1980 to 2004 and reported the pooled prevalence as 2.8% (95% CI: 2.5–3.1) in the population aged 60 and older ranging from 0.8% to 6.1% [4]. In Taiwan, the range of prevalence in the previous 8 studies since 1980 was from 2.7% to 4.4% [6]. In Hong Kong, the prevalence in the people aged 70 and over was estimated to increase from 4.5% to 9.3% since 1995 [7]. A recent review which included 48 studies in mainland China, Hong Kong and Taiwan from 1980 to 2010 provided a pooled estimate for the population aged 60 and over as 3.0% (95% CI: 2.4–3.9), ranging from 0.6–9.0% across age-bands, and reported higher prevalence in northern China (3.9%, 95% CI: 2.8–5.5) than in southern China (2.9%, 95% CI: 1.9–3.6) [5]. However, methods of research and study design, which vary greatly between the different studies, were not considered. These large differences cause uncertainty in producing reliable summary estimates for nationwide and different area prevalence. Without considering the difference of study methods, area variations of dementia prevalence cannot be examined appropriately. It is important to explore the impact that methodologies might have in order to produce estimates that adjust of these differences. This paper describes a detailed investigation of Chinese dementia prevalence studies including specific study design and methodological properties for mainland China, Hong Kong and Taiwan and estimates as robustly as possible the current and future numbers of people with dementia across mainland China, Hong Kong and Taiwan.

## Methods

### Literature Search

Relevant literature in Chinese (both simplified and traditional characters) and English was identified through an electronic search of three English and two Chinese databases: PubMed, ScienceDirect, PsycInfo Chinese National Knowledge Infrastructure (CNKI) and Airti Library. The databases were searched from their inception until April 2012. A protocol was developed for the review and the PRISMA systematic review guidelines were followed [8]. Search strategy details are included in (S2 in File S1).

The following inclusion criteria were used to select papers: (1) Cases were collected by field survey, not based on hospital data. (2) The study involved a population sampling rather than recruiting volunteer participants. (3) The study reported the prevalence amongst people aged 50 and over. (4) Dementia case status was not decided only by a screening test and the specific instruments and criteria for case identification were reported. Studies which focused on the Chinese population outside mainland China, Hong Kong and Taiwan were excluded.

### Data Extraction

Details of each study were extracted systematically based on a form designed by the authors (S1 in File S1). The data extraction form included four sections which detailed methodological factors and the characteristics of study population:

1. Study design: methods of screening, diagnosis and confirmation, interviewers and sampling method;
2. Participants: sample size and response rate, characteristics of participants, such as age group, study location, urban or rural area;
3. Dementia identification: screening tools, diagnostic criteria and instruments;
4. Results: overall prevalence of all type dementia and stratified prevalence by age, gender and educational level.

Two readers reviewed all the articles and filled out the questionnaires independently. All the information in each study was double verified and confirmed by the two readers. Any discrepancies between the readers’ extracted data were reconciled through discussion and one final consensus decision agreed.

### Statistical Analysis

Meta-analysis was used to calculate the overall and area level pooled estimate of dementia prevalence. The provinces and cities in mainland China were categorised into 3 large geographical areas: north, central, south. North China included Beijing, Liaoning, Heilongjiang, Shandong, Hebei, Henan, Shanxi, Shaanxi, Gansu and Xinjiang. Central China included Shanghai, Chongqing, Jiangsu, Zhejiang, Anhui, Hubei, Hunan, Jiangxi and Sichuan. South China included Fujian, Guizhou, Guangdong and Hainan. Pooled prevalence was calculated by random-effect estimation due to the large heterogeneity across the studies [9]. The studies were divided into three socioeconomic levels based on both economic (average household income of the province by urban and rural areas) and political status (municipality, city or county). The studies in Hong Kong and Taiwan were combined in one group. Cross-province studies were separated from above geographical areas and categorised in one group.

Meta-regression was conducted to explore whether the heterogeneity can be explained by some of the methodological factors or the characteristics of study populations in univariate and multivariate models [10]. The change of I^2^, an indicator of residual variation across studies, was used to measure the effect of variables on explaining heterogeneity. Since there was large variation in age group categories between studies, age standardisation was conducted to adjust for the effect of age structure on dementia prevalence. All the studies, including the surveys in Hong Kong and Taiwan, were standardised to the Census Population of China 2010 [11].

### Estimation of Numbers of People with Dementia

In order to estimate the number of people with dementia taking methodological factors and area differences into account, the age-stratified prevalence was generated based on the results of meta-regression and the assumption of doubling prevalence with each five year increase in age from which there has been almost no deviation in any study of dementia across the world- irrespective of starting prevalence. The stratified prevalence by 5-year age groups from 60 years old in northern, central and southern areas of mainland China was calculated in the same diagnostic criteria, DSM-IV, which is a newer standard of dementia diagnosis. Since most of the studies in Hong Kong and Taiwan included their participants from the age of 65 years, it is more appropriate to use the estimated prevalence in the population aged 65 and over in meta-regression and generate 5-year age-stratified prevalence starting from 65. In terms of public health implication, Hong Kong and Taiwan are more likely to consider older population as people aged 65 and over. Therefore, the age range of estimated prevalence and numbers was slightly different in these three areas. The gender ratio of prevalence, which was found in the meta-analysis, was used to generate the approximate age-stratified prevalence by men and women.

The age-stratified prevalence was applied to the population in different areas, provinces and cities to estimate the number of people with dementia. Information on population estimates were obtained from the sixth Nation Population Census in 2010 by National Bureau of Statistics of China, official statistics of Department of Interior, Taiwan and Census and Statistics Department, Hong Kong Special Administrative regions [11]–[14]. In mainland China, there are eight provinces (Jilin, Inner Mongo, Tibet, Qinghai, Ningxia, Guangxi, Guizhou and Yunnan) and one city (Tianjin) without existing prevalence studies in the area. The model of North China was applied to the provinces in northern (Jilin and Tianjin) and western (Inner Mongo, Tibet, Qinghai, Ningxia) areas and the model of South China was applied to the other three provinces (Guangxi, Guizhou and Yunnan) based on their geographical locations. Similar methods were used to project the number of people with dementia in the next 50 years. The estimated population structures of mainland China was obtained from World Population Prospects, the 2010 version. For Taiwan and Hong Kong, the data was obtained from official statistics of Department of Interior, Taiwan and Census and Statistics Department, Hong Kong Special Administrative regions (SAR). Details of estimate method are provided in S2 in File S1.

## Results

**Figure 1:**
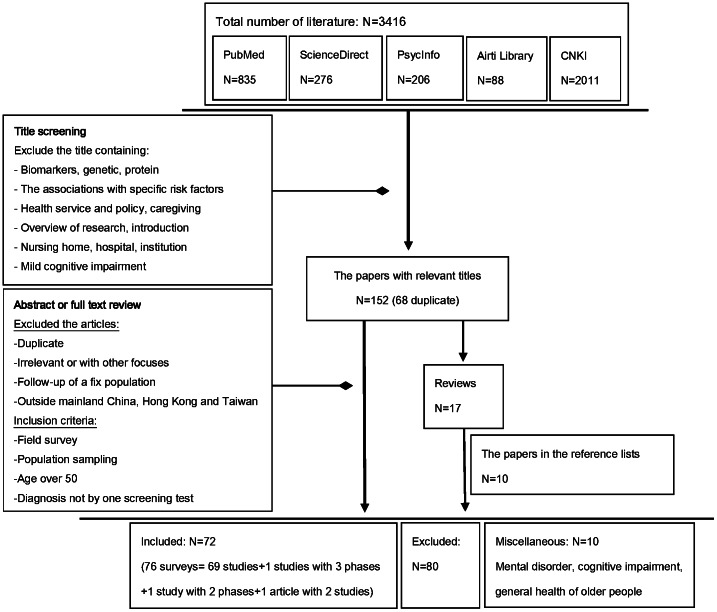
Flow plot of literature search.

Figure 1 shows a flow chart of the number of studies identified, screened and included in the review. In total, 72 papers, reporting the findings from 76 surveys, met the inclusion criteria: 69 reported the findings of a single survey, 1 reported a two-phase survey, 1 reported a three-phase survey and 1 reported two separate surveys. The list of the identified studies was provided in S2 in File S1.

### Descriptive Statistics

The 76 studies covered 19 provinces, 3 municipalities and 2 nationwide studies in mainland China, 6 studies in Taiwan and 1 study in Hong Kong [15]–[86]. There were 25 surveys (32.9%) conducted in North China [15]–[39], 26 (34.2%) in central area [40]–[62] and 16 (21.1%) in southern area [63]–[78]. The 2 nationwide studies were the 4 Cities Study and the 4 Provinces Study [60], [79]. The 4 Cities Study started from 1997–1998 and recruited people aged over 55 years old in 4 main cities across China: Beijing, Xian, Shanghai and Chengdu [79]. The 4 Provinces Study investigated people over 60 years old in Shanghai, Shanxi, Guangdong and Heilongjiang provinces in 2008–2009 [60]. The 6 studies conducted in Taiwan included 3 surveys in southern Taiwan (KaoKaoping area and Kinmen), 2 surveys in northern area (Taipei and Ilan) and 1 nationwide study (4 geographical regions: north, central, south and east) [81]–[86].

Diagnostic criteria used in the 76 studies included the Diagnostic and Statistical Manual of Mental Disorder Third and Fourth Edition (DSM-III, -IV), the International Statistical Classification of Diseases 9^th^ and 10^th^ (ICD-9, -10), Chinese Classification of Mental Disease (CCMD), 10/66 algorithm and Geriatric Mental State Examination- Automated Geriatric Examination for Computer Assisted Taxonomy (GMS-AGECAT).

### Pooled Prevalence

**Figure 2:**
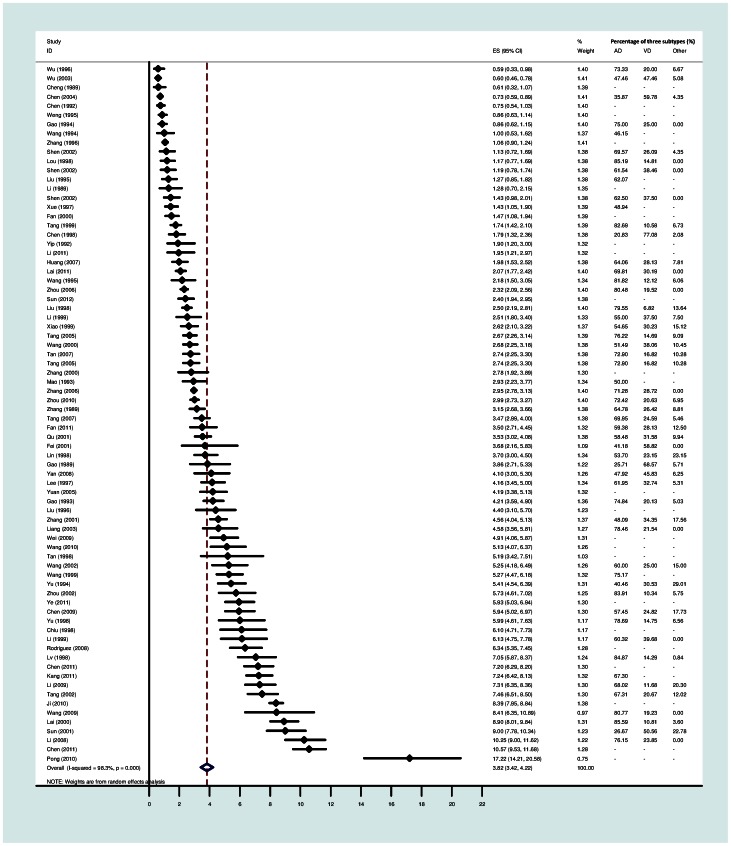
Forest plot of the prevalence studies of dementia in mainland China, Hong Kong and Taiwan.

The pooled prevalence of dementia from the 76 studies was 3.8% (95% CI: 3.4, 4.2) with the range from 0.6% to 17.2% (Figure 2). The average percentage of three subtypes, Alzheimer’s disease, vascular dementia and other types were 63.3%, 28.5% and 7.5% respectively. The overall estimate from the 70 surveys with age standardisation was 3.9% (95% CI: 3.5, 4.3). The heterogeneity in these studies was extremely high (I-squared = 98.3%), meaning that over 98% of residual variation was attributed to heterogeneity. After age standardisation, there is little change in the I-squared.

**Figure 3:**
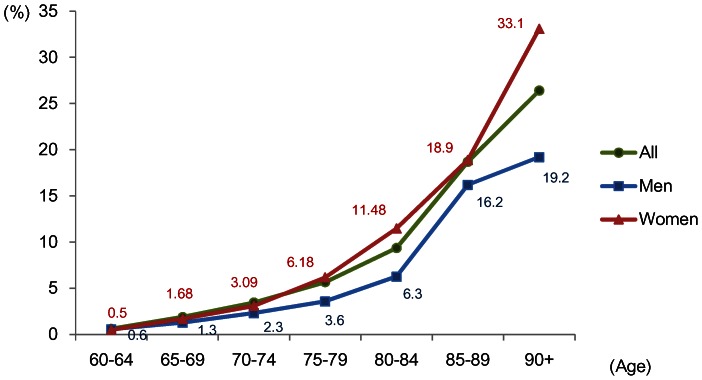
Stratified prevalence by age groups and gender.

Based on available data in the studies, the pooled estimates of stratified prevalence by 5-year age groups and gender are presented in Figure 3. The pooled prevalence of dementia by age groups 60–64, 65–69, 70–74, 75–79, 80–84, 85–89, 90 and above was 0.6% (95% CI: 0.4, 0.8), 1.9% (95% CI: 1.5, 2.3), 3.5% (95% CI: 2.8, 4.1), 5.7% (95% CI: 4.7, 6.7), 9.4% (95% CI: 8.0, 10.7), 18.7% (95% CI: 15.8, 21.6) and 26.4% (95% CI: 21.2, 31.6) respectively. The pattern of increased prevalence approximately doubling every 5 years is seen. Women had higher prevalence than men across all age groups above age 65. The pooled prevalence in North China was 4.8% (95% CI: 4.0, 5.7). Central and South China had lower prevalence: 3.2% (95% CI: 2.5, 4.0) and 3.2% (95% CI: 2.5, 3.8) respectively. The pooled prevalence in Hong Kong and Taiwan was 3.3% (95% CI: 2.3, 4.2).

### Meta-regression

The results of the meta-regression are presented in Table 1. The overall estimate for age-standardised prevalence was 3.8% (95% CI: 2.6, 4.9) in Model 1 which controlled for the effect of diagnostic criteria, age group, sampling method, population size and geographical areas. The prevalence of dementia significantly decreased from northern, central to southern areas of China in standardised and adjusted model. The pooled prevalence in Central China was 1% lower than the northern areas although this difference was not statistically significant. The overall estimate for the southern areas was around 2% lower than the northern areas while in Hong Kong and Taiwan, the larger difference compared to North China was over 3%. The associations remained significant after further adjusting for the socioeconomic level of the study area, a measure based on economic development and political status of the provinces and cities (Model 2). It indicated that the area variation of dementia prevalence did not be explained by socioeconomic level of area.

**Table 1:** Multivariable analysis (standardised to the census population of China 2010).

			Model 1				Model 2		
Category		Coeff.	95% CI	p-value	F-test	Coeff.	95% CI	p-value	F-test
**Overall**		3.75	(2.57, 4.93)	<0.001	<0.001	3.91	(2.67, 5.14)	<0.001	<0.001
**Area**	North (ref.a.)	–	–	–	0.011	–	–	–	0.005
	Central	−1.14	(−2.37, 0.08)	0.07		−1.28	(−2.47, −0.09)	0.04	
	South	−1.89	(−3.28, −0.49)	0.01		−1.76	(−3.12, −0.40)	0.01	
	Hong Kong and Taiwan	−3.34	(−5.29, −1.38)	0.001		−3.65	(−5.64, −1.67)	0.001	
	Nationwide	−0.11	(−3.16, 2.94)	0.94		−0.23	(−3.19, 2.74)	0.88	
**Diagnostic**	DSM-III/–III-R (ref. a.)	–	–	–	0.006	–	–	–	0.005
**criteria** d.	DSM-IV/–IV-R	1.69	(0.53, 2.84)	0.004		1.69	(0.58, 2.80)	0.004	
	ICD-10	−1.30	(−3.67, 1.07)	0.28		−1.55	(−3.89, 0.79)	0.19	
	CCMD	−1.09	(−4.35, 2.16)	0.55		−0.41	(−3.65, 2.83)	0.80	
	Mixed	1.21	(−0.33, 2.74)	0.14		0.88	(−0.63, 2.40)	0.25	
	Other	3.02	(0.68, 5.36)	0.012		2.86	(0.57, 5.16)	0.02	
**Whole study age**	50+	−1.47	(−4.16, 1.22)	0.28	0.001	−0.78	(−3.47, 1.90)	0.56	0.003
**range**	55+	0.09	(−1.30, 1.48)	0.90		0.19	(−1.15, 1.53)	0.77	
	60+ (ref. a.)	–	–	–		–	–	–	
	65+	2.38	(1.13, 3.63)	0.001		2.76	(1.49, 4.04)	<0.001	
	70+	3.97	(0.97, 6.96)	0.01		4.06	(1.15, 6.97)	0.01	
**Sampling method**	Place (ref. a.)	–	–	–	0.017	–	–	–	0.021
b.	People	1.88	(0.52, 3.23)	0.01		1.72	(0.41, 3.03)	0.01	
	Unknown	−1.07	(−4.39, 2.26)	0.52		−1.30	(−4.57, 1.97)	0.43	
**Study size**	Less than 5000 (ref. a.)	–	–	–	0.007	–	–	–	0.004
	More than 5000	−1.75	(−2.99, −0.51)	0.01		−1.82	(−3.03, −0.61)	0.004	
**Socioeconomic**	High (ref. a.)					–	–	–	0.065
**level of area** c.	Medium					0.94	(−0.46, 2.33)	0.19	
	Low					−0.77	(−1.91, 0.37)	0.18	
**I-square**		95.1%				94.6%			

a. Ref.: reference group.

b. Sampling method: if the study included all the older people in the specific geographical areas, it was considered as “place” type of sampling unit. If the final sampling unit of the study was each individual, it was categorised as “people” type.

c. Socioeconomic level of area: based on economic development information (average household income) and political status (municipality, city and county) of the provinces or areas in China Statistical Yearbook 2010 [10]. For Hong Kong and Taiwan, the two nationwide studies were separated as two categories and dropped because of collinearity.

d. Diagnostic criteria: DSM-III/–IV: Diagnostic and Statistical Manual of Mental Disorder Third/Fourth Edition; ICD-9/10: the International Statistical Classification of Diseases 9th/10^th^; CCMD: Chinese Classification of Mental Disease; Mixed: multiple diagnostic criteria including DSM, ICD or CCMD.

Diagnostic criteria, whole study age range, population size and sampling method were significantly associated with the dementia prevalence observed in the individual studies. Compared to studies using DSM-III or DSM-III-R diagnostic criteria, studies using DSM-IV or DSM-IV-R criteria had about 1.7% higher prevalence and other methods, including 10/66 and GMS-AGECAT that were grouped due to low frequency, showed about 3% higher prevalence. As expected there was a steady increase in prevalence with age, studies that included only participants aged 65 years and above had 2.4% higher prevalence, and only aged 70 years and above had 4.0% higher prevalence than age 60 and above group. The estimated prevalence of studies with a sample size above 5000 was 1.8% lower than smaller studies. Sampling method focused on the final sampling unit and divided into two types: by place and by people. Studies whose final sampling unit was by place, where participants were recruited in specific geographical units using clustered sampling, had about 2% lower pooled prevalence than studies that further sampled individuals in study areas. The effect of gender ratio was examined in both univariable and multivariable models but it was not significantly associated with differing dementia prevalence. Several factors of study design, such as sampling negative case in screening test for diagnosis, the types of interviewer and location of investigation, were examined in regression models but none of them had significant influence on dementia prevalence.

### Estimated Number of People with Dementia

**Figure 4:**
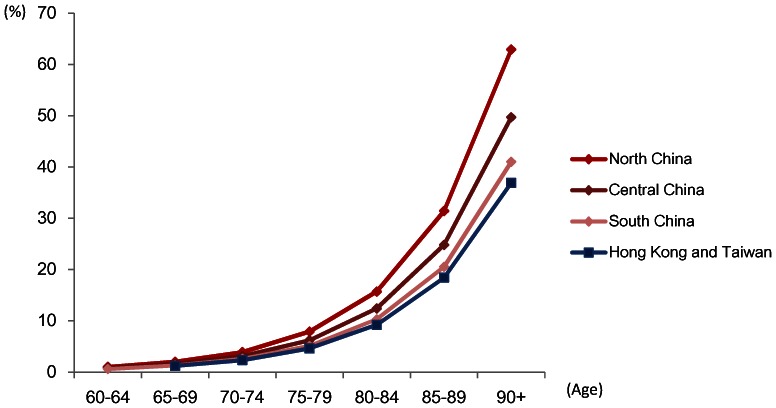
Estimated age-stratified prevalence by north, central, south of mainland China, Hong Kong and Taiwan (Based on DSM-IV). ^a.^ Age range of estimation: The estimates of three areas in mainland China started from 60 years old, which is the definition of older people in People’s Republic of China. Both Hong Kong and Taiwan are more likely to consider elderly population as people aged 65 and above since they have higher proportion of ageing population since early 1990s. Most of their prevalence studies of dementia also recruited the participants aged 65 and over. Therefore, it is more reliable and reasonable to estimate age-stratified prevalence from 65 years old.

**Figure 5:**
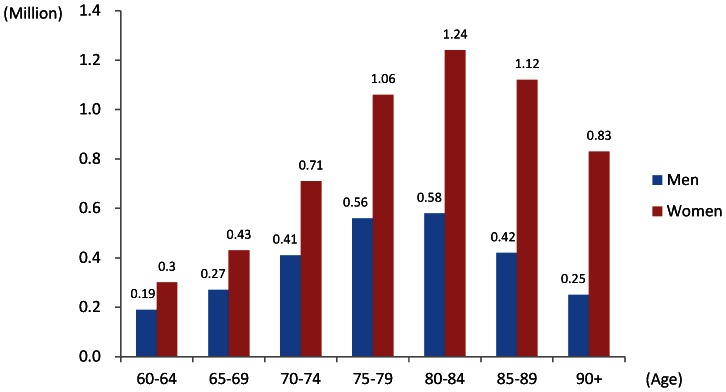
Estimated number of people with dementia by age and gender.

The age-stratified prevalence based on the newer standard of diagnosis, DSM-IV, in different areas is demonstrated in Figure 4. The estimated prevalence was expected to be higher than the results in meta-regression model, which used the major type of diagnostic criteria DSM-III as baseline. Total numbers of people with dementia by age groups and gender in mainland China, Hong Kong and Taiwan are provided in Figure 5. Based on the diagnostic criteria of DSM-IV, it is estimated that 8.4 million (4.6%, 95% CI: 3.4, 5.8) people aged 60 and over have dementia in these three areas. 5.3 million (63%) of them are women and 3.1 million are in men. Nearly 40% (3.4 million) of the demented population mainly concentrates in the age range 75 to 84 and 30% (2.6 million) in age over 85.

**Figure 6:**
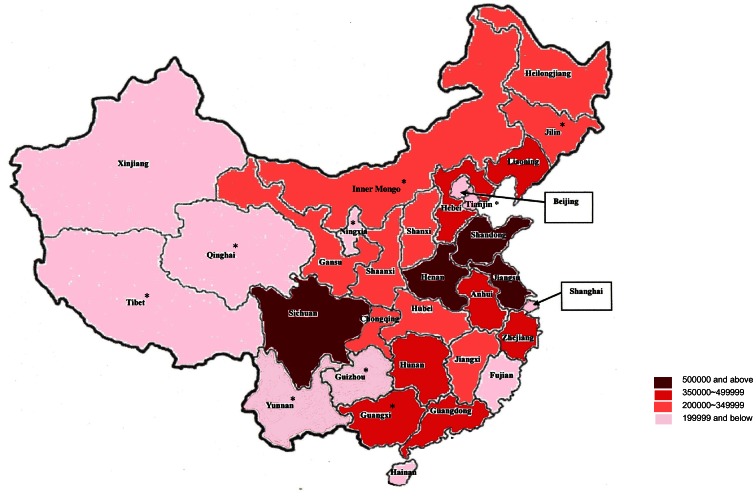
Estimated numbers of people with dementia by provinces and cities. * The province without existing study; Jilin, Tianjin, Inner Mongo, Ningxia, Qinghai, Tibet were estimated by the model of North China. Guizhou, Yunnan and Guangxi were estimated by the model of South China.

The estimated numbers of people with dementia by areas are provided in Table 2. The numbers of people aged 60 and over with dementia in north, central and south of mainland China are 3.8 (5.1%, 95% CI: 4.1, 6.1), 3.2 (4.4%, 95% CI: 3.2, 5.6), and 1.2 (3.9%, 95% CI: 2.3, 5.4) million respectively. In Hong Kong and Taiwan, 0.21 million of the population aged 65 and over are estimated to have dementia. Detailed estimations by provinces and cities in mainland China are presented in Figure 6. The range is from 5.7% (Shandong) to 3.1% (Guizhou). High prevalence numbers are estimated for developed areas, such as municipalities (Beijing and Shanghai) and the provinces near the east coast. More detailed estimates by each province are provided in (S3 in File S1).

**Table 2:** Estimated number of people with dementia in mainland China, Hong Kong and Taiwan (Based on DSM-IV).

Area		Total population (million)	Elderly population a.(60+ in mainland China; 65+ inHong Kong and Taiwan, million)	Number of people with dementia (million)	Prevalence b.(%, 95% CI)
**Mainland China**	North	563.8	73.4	3.76	5.1 (4.1, 6.1)
(age 60+) a.	Central	492.4	73.3	3.22	4.4 (3.2, 5.6)
	South	276.6	30.9	1.20	3.9 (2.3, 5.4)
	Total	1332.6	177.6	8.18	4.6 (3.4, 5.8)
**Hong Kong** (age 65+) a.		6.9	0.9	0.06	6.8 (3.8, 9.8)
**Taiwan** (age 65+) a.		23.2	2.5	0.15	5.7 (3.2, 8.2)
**Total**		1362.92	181	8.39	4.6 (3.4, 5.8)

a. Since most of their prevalence studies of dementia in Hong Kong and Taiwan recruited the participants aged 65 and over, it is more reasonable to estimate age-stratified prevalence from 65 years old and apply these estimates to the population aged 65 and over.

b. The prevalence in mainland China considered the people aged 60 and above while in Hong Kong and Taiwan, the prevalence focused on the population aged 65 and over.

The projected number of people with dementia in next 50 years by mainland China, Hong Kong and Taiwan is provided in Table 3. With population ageing the numbers of people with dementia are estimated to increase substantially and double every 20 years. The considerable population with dementia in mainland China is expected to reach 20 million by 2030 and exceed 40 million by 2050.

**Table 3:** Projected number of people with dementia from 2012 to 2060 in mainland China, Hong Kong and Taiwan (Based on DSM-IV, unit: million).

	Current	2020	2030	2040	2050	2060
**Mainland China**(age 60+)	8.18	13.5	20.3	29.59	40.63	48.68
**Hong Kong**(age 65+)	0.06	0.09	0.13	0.20	0.29	0.32
**Taiwan**(age 65+)	0.15	0.21	0.32	0.49	0.66	0.71

## Discussion

### Main Findings

The overall estimate of dementia prevalence in mainland China, Hong Kong and Taiwan was 3.8% (95% CI: 2.6, 4.9) after controlling for methodological factors and geographical areas. Of the many designs and methodological influences explored, the most important were diagnostic criteria, whole study age range, population size and sampling method which influenced estimates. A significant decreasing pattern from north, central, south areas of mainland China to Hong Kong and Taiwan was found.

The results of the meta-regression model were used to estimate the numbers of people with dementia in different cities or provinces. Based on diagnostic criteria DSM-IV, The estimated number of older people with dementia in mainland China, Hong Kong and Taiwan is 8.4 million (4.6%, 95% CI: 3.4, 5.8). High prevalence numbers are more likely to concentrate in metropolitan cities and the provinces near east coast.

### Strength and Limitations

This review collected prevalence studies in mainland China, Hong Kong and Taiwan published in either English or Chinese from 1980 to 2012. Compared to previous reviews, this study included more recent surveys which used newer diagnostic methods (such as DSM-IV, 10/66 and GMS-AGECAT) and reported slightly higher pooled prevalence, nearly 4% [2], [5]. Key methodological factors and characteristics of study population were extracted from each study. The effects of survey methods were controlled for in a meta-regression analysis to estimate the overall and area prevalence of dementia and the results were applied to national demographics to calculate the numbers of people with dementia by areas.

Most of the studies with more rigorous and comparable study designs concentrated in highly developed areas, such as Beijing, Shanghai, Guangdong and the east coast. Few surveys had been conducted in western areas, nor in the north-east and south-west boarder provinces. The unbalanced distribution of the studies limits our ability to assess variation between western and eastern areas and to estimate reliably the numbers of people with dementia in western areas. Only about 16% of studies were conducted in rural areas while 40% were in urban areas and the rest were categorised as mixed type. There was no significant variation between these three groups after age standardisation. However, the definition of urban and rural area is complicated in mainland China. In this review, the category of rural and urban area was based on the description in each study. A city or county can involve both rural and urban areas and it is difficult to define a study as urban or rural only according to its location. More information is needed to investigate potential variation through multicentre studies with identical designs (China Cognition and Ageing Study, Alzheimer’s Association International Conference 2012 abstract).

### Insufficient and Incomplete Information

The quality of the research papers was rather variable. Insufficient and uncertain data caused difficulties in controlling for the many potential influences which might result in heterogeneity between studies and examining possible relationships between the variables. Moreover, several studies did not report stratified prevalence by 5-year age groups. The small sample size in each age group also limited our ability to explore the associations between covariates and prevalence in specific age groups. It was necessary to construct a method to infer the stratified prevalence and the differences in the estimated prevalence between various areas were magnified in the older age groups. Especially in the last two age groups (85–89, 90+), the estimates might vary largely from crude prevalence. However, study populations in these age groups are usually much smaller and estimates are liable to be unreliable.

Higher prevalence might be expected in Hong Kong and Taiwan in line with western estimates since both had earlier economic development and demographic changes than mainland China. However, in the regression model, the prevalence of dementia in Hong Kong and Taiwan is estimated to be about 3% lower than in the northern area of mainland China. This might be attributed to the small sample (7 studies in Hong Kong and Taiwan). Furthermore, diagnostic criteria and sampling units used in studies across the four geographical areas also varied. The potential interaction terms were once considered in the model but there was not sufficient information in data to investigate them. This paper has not included incidence and mortality as these do not yet have sufficient data of robust quality for analysis.

### Miscellaneous Studies

Several studies that investigated general mental disorders including dementia were excluded or categorised as miscellaneous because of their ambiguous screening methods and diagnostic criteria. It is expected that the processes of case identification in these surveys were different from the studies which only focus on dementia. Most of them reported low prevalence of dementia (<1%) in the population aged 60 years and above. Standardised diagnosis of mental illness in mainland China developed in the early 1980s and the process of investigation and diagnosis varies across surveys. It is almost impossible to compare these results with the included studies. Qualitative papers were revealing in this regard, including negative descriptions about symptoms of senile dementia, giving a clue of the stigma associated with dementia in early 1980s and the under-reporting of cases and subsequent risk of bias [87].

### Implications for Future Discipline Studies

Geographical variations in dementia prevalence do suggest variation worthy of further study. Some previous studies also reported the variation of crude prevalence between northern and southern areas [5], [79]. In this review, the findings provided an estimate of area differences taking the effect of different survey methods and age structures into account. However, extremely high heterogeneity (over 90%) indicates large unexplained variations behind the pooled prevalence. There may be other design and implementation measures, such as sampling scheme, quality of interviews, which are not reported but do affect results and only the way to examine this would be much improved study reporting. To obtain more reliable estimates of dementia prevalence, these factors need to be improved and controlled for in future primary and secondary research.

The variation across large areas of China was not explained by many methodological factors and socioeconomic level of areas. Differences in life expectancy between cities and provinces does not explain geographical variation through residual age confounding since it did not correspond the decreasing pattern from north to south. Variations between areas in education, lifestyle, living environment and health care may explain geographical differences in dementia prevalence. Within mainland China, there are considerable area variations of natural and social environment with differing lifestyle and customs from north to south and between ethnic groups. Furthermore, mainland China, Hong Kong and Taiwan all have very different histories, political and economic development, which can affect social environment, education system, living conditions and the life experiences of older people.

There have been two nationwide studies, the 4 Cities Study and 4 Province Study, which investigated the prevalence of dementia in different places with the same research methods [60], [79]. 4 Cities Study reported some variations between different cities and 4 Provinces Study found significantly higher risk of dementia in rural than in urban areas. A preliminary result of a recent study in mainland China also reported higher prevalence in rural areas. One implication of these findings may be that they indicate the potential effects of environment on prevalence of dementia. To understand the factors which cause geographical variations, a multicentre study would be needed with a sufficiently large sample, good response rate representative of the national population of older people, consistent design, strict quality control and rolling surveillance with 5 year annual replenishment of the youngest cohort and migration. In addition, it would be informative to explore variation in potential risk factors which profiles across the mid-life population to examine their role.

The application of the results in meta-regression to national population data suggests the best estimate of people with dementia in East Asia region and reveals the areas in mainland China which have the greatest challenge. Metropolitan cities (Beijing, Shanghai and Chongqing) have substantial number of people with dementia. Four provinces, Shandong, Henan, Jiangsu and Sichuan, are estimated to contain a considerable population with dementia of over 2.5 million. Although the numbers of people with dementia in Hong Kong and Taiwan are much smaller compared to mainland China, the prevalence is relatively high (6.8% and 5.6%) because of their age structures. The distribution across age groups indicates the bulk is in the 75 to 89 age range. This means that the current majority of dementia patients are in these slightly older age groups and also indicates large numbers of people in the oldest old age groups about which very little are known [88]. In the future, this is likely to shift to increasing numbers in 85 and above age group in which needs and demands on society are different. These estimates should assist in considering nationwide policies for research and science developed to address the needs of the population in different areas in mainland China, Hong Kong and Taiwan.

As a substantial public health issue, dementia not only intensifies the challenges of ageing societies but also raises awareness of mental health in the oldest old. The results of this review suggest important clues to explore geographical variations in dementia and the potential risk factors in area level which are influential to the health of cognition and mood in the older population. These robust estimates of the population with dementia provide important evidence to indicate the various needs in different areas and support nationwide and local policy making of mainland China, Hong Kong and Taiwan.

## Supporting Information

File S1 **S1–S3.** S1. Data extraction form S2. Included, excluded and miscellaneous studies S3. Estimating the number of people with dementia in mainland China, Hong Kong and Taiwan
(DOCX)
