# Supplementary material for: Prevalence Studies of Dementia in Mainland China, Hong Kong and Taiwan: A Systematic Review and Meta-Analysis
Source: PLoS One. 2013 Jun 11;8(6):e66252. doi: 10.1371/journal.pone.0066252 (PMC3679068; doi:10.1371/journal.pone.0066252)
Supplement: File S1 — S1–S3. S1. Data extraction form S2. Included, excluded and miscellaneous studies S3. Estimating the number of people with dementia in mainland China, Hong Kong and Taiwan (DOCX) [file pone.0066252.s001.docx]

**Prevalence studies of dementia in mainland China, Hong Kong and Taiwan: a systematic review and meta-analysis**

**Supporting information**

S1. Data extraction form

S2. Included, excluded and miscellaneous studies

S3. Estimating the number of people with dementia in mainland China, Hong Kong and Taiwan

**S1. Data extraction form**

Systematic Review of dementia in mainland China, Hong Kong and Taiwan: **Prevalence study**

**Id: _______ Authors: ____________ Year: _________ Location: ____________**

**1. Design:**

1.1 Phases of investigation

| Phase (year) | | | Executor | | Method | | | | Proportion to next stage | | | Others |
| --- | --- | --- | --- | --- | --- | --- | --- | --- | --- | --- | --- | --- |
| I |  | Screening |  | Interviewer  ( ) |  | Face-to-Face |  | Home |  | All positive | |  |
|  |  |  |  |  |  | Self-completed |  | Community |  | + negative ( ) | |  |
|  |  |  |  | Medical Prof.  ( ) |  | Informant |  | Hospital |  | +suspected ( ) | |  |
|  |  |  |  |  |  |  |  |  |  |  | |  |
|  |  | Diagnosis |  | Interviewer  ( ) |  | | | |  | | |  |
|  |  |  |  | Medical Prof.  ( ) |  |  |  |  |  |  |  |  |
|  |  |  |  |  |  | | | |  | | |  |
| II |  | Screening |  | Interviewer  ( ) |  | Face-to-Face |  | Home |  | | All positive |  |
|  |  |  |  |  |  | Self-completed |  | Community |  | | + negative ( ) |  |
|  |  |  |  | Medical Prof.  ( ) |  | Informant |  | Hospital |  | | +suspected ( ) |  |
|  |  |  |  |  |  |  |  |  |  | |  |  |
|  |  | Diagnosis |  | Interviewer  ( ) |  | | | |  | | |  |
|  |  |  |  | Medical Prof.  ( ) |  |  |  |  |  |  |  |  |
|  |  |  |  |  |  | | | |  | | |  |
| III |  | Screening |  | Interviewer  ( ) |  | Face-to-Face |  | Home |  | All positive | |  |
|  |  |  |  |  |  | Self-completed |  | Community |  | + negative ( ) | |  |
|  |  |  |  | Medical Prof.  ( ) |  | Informant |  | Hospital |  | +suspected ( ) | |  |
|  |  |  |  |  |  |  |  |  |  |  | |  |
|  |  | Diagnosis |  | Interviewer  ( ) |  | | | |  | | |  |
|  |  |  |  | Medical Prof.  ( ) |  |  |  |  |  |  |  |  |
|  |  |  |  |  |  | | | |  | | |  |
| IV |  | | | |  | | | |  | | |  |

1.2 Sampling

|  | Convenience sampling |  | Simple random |  | Systematic sampling |
| --- | --- | --- | --- | --- | --- |
|  | Stratified sampling |  | Cluster sampling |  | Quota sampling |
|  | Probability proportional |  | Unknown |  |  |
|  |  |  |  |  |  |
| Final sampling unit: | |  | Place |  | People |

Stratified layers and clusters:

|  | Units in the layer | Selected unit | Random |
| --- | --- | --- | --- |
| Layer 1 |  |  |  |
| Layer 2 |  |  |  |
| Layer 3 |  |  |  |
|  |  |  |  |
| Final |  | |  |
| Others |  | | |

**2. Participants**

2.1 Number and response rate

| Phase (Year) | | Number | | Response rate | Drop out | | | | Others |
| --- | --- | --- | --- | --- | --- | --- | --- | --- | --- |
|  |  | Expected | Actual |  | Death | Case | Reject/ Lost | Age/Gender/Area |  |
| I | 1 |  |  |  |  |  |  |  |  |
|  | 2 |  |  |  |  |  |  |  |  |
|  | 3 |  |  |  |  |  |  |  |  |
| II | 1 |  |  |  |  |  |  |  |  |
|  | 2 |  |  |  |  |  |  |  |  |
|  | 3 |  |  |  |  |  |  |  |  |
| III | 1 |  |  |  |  |  |  |  |  |
|  | 2 |  |  |  |  |  |  |  |  |
|  | 3 |  |  |  |  |  |  |  |  |
|  |  |  |  |  |  |  |  |  |  |

2.2 Characteristics:

| Sample size, age | Nationwide/Province/ City | County/District/ Town | | Urban/ Rural/Mixed | Other |
| --- | --- | --- | --- | --- | --- |
|  |  |  | |  |  |
| Age structure | | Gender, Number (%) | | Education | |
| Group | Number (%) | Men | Women | Group | Number (%) |
|  |  |  |  |  |  |
|  |  |  |  |  |  |
|  |  |  |  |  |  |
|  |  |  |  |  |  |
|  |  |  |  |  |  |
|  |  |  |  |  |  |

**3. Dementia identification**

3.1 Screening:

| Instruments | Cut-off points for positive case/ description | | | | | | Others |
| --- | --- | --- | --- | --- | --- | --- | --- |
|  | Phase 1 | | Phase 2 | | Phase 3 | |  |
| MMSE |  |  |  |  |  |  |  |
| MMSE-C |  |  |  |  |  |  |  |
| ADL |  |  |  |  |  |  |  |
| BSSD |  |  |  |  |  |  |  |
| CRBRS |  |  |  |  |  |  |  |
| CSI-D |  |  |  |  |  |  |  |
| HDS |  |  |  |  |  |  |  |
| 10/66 |  |  |  |  |  |  |  |
| Medical history |  |  |  |  |  |  |  |
| Unknown |  |  |  |  |  |  |  |
|  |  |  |  |  |  |  |  |
|  |  |  |  |  |  |  |  |
|  |  |  |  |  |  |  |  |

3.2 Diagnosis

| Criteria/ Instruments | Version | | | | | | Others |
| --- | --- | --- | --- | --- | --- | --- | --- |
|  | Phase 1 | | Phase 2 | | Phase 3 | |  |
| ADL |  |  |  |  |  |  |  |
| BDS |  |  |  |  |  |  |  |
| CCMD |  |  |  |  |  |  |  |
| CDR |  |  |  |  |  |  |  |
| DSM |  |  |  |  |  |  |  |
| FOM |  |  |  |  |  |  |  |
| HIS |  |  |  |  |  |  |  |
| ICD |  |  |  |  |  |  |  |
| NINCDS-ADRDA |  |  |  |  |  |  |  |
| NINDS-AIREN |  |  |  |  |  |  |  |
| POD |  |  |  |  |  |  |  |
| WAIS |  |  |  |  |  |  |  |
| DS |  |  |  |  |  |  |  |
| RVR |  |  |  |  |  |  |  |
| WISC |  |  |  |  |  |  |  |
| HAMD |  |  |  |  |  |  |  |
| Unknown |  |  |  |  |  |  |  |
|  |  |  |  |  |  |  |  |
|  |  |  |  |  |  |  |  |

**4. Results**

4.1 By age (Prev. : Prevalence, %; S/C: standardised or Crude)

**Standardised population:**

| All types | | | | | AD | | | | VD | | | |
| --- | --- | --- | --- | --- | --- | --- | --- | --- | --- | --- | --- | --- |
| Age | Case | Population | Prev. | S/C | Case | Population | Prev. | S/C | Case | Population | Prev. | S/C |
|  |  |  |  |  |  |  |  |  |  |  |  |  |
|  |  |  |  |  |  |  |  |  |  |  |  |  |
|  |  |  |  |  |  |  |  |  |  |  |  |  |
|  |  |  |  |  |  |  |  |  |  |  |  |  |
|  |  |  |  |  |  |  |  |  |  |  |  |  |
|  |  |  |  |  |  |  |  |  |  |  |  |  |
|  |  |  |  |  |  |  |  |  |  |  |  |  |
|  |  |  |  |  |  |  |  |  |  |  |  |  |
|  |  |  |  |  |  |  |  |  |  |  |  |  |
|  |  |  |  |  |  |  |  |  |  |  |  |  |
|  |  |  |  |  |  |  |  |  |  |  |  |  |
|  |  |  |  |  |  |  |  |  |  |  |  |  |
|  |  |  |  |  |  |  |  |  |  |  |  |  |

4.2 By gender (Prev. : Prevalence; S/C: standardised or Crude)

|  | | Male | | | | Female | | | |
| --- | --- | --- | --- | --- | --- | --- | --- | --- | --- |
| Type | Age | case | Population | Prev. | S/C | case | Population | Prev. | S/C |
|  |  |  |  |  |  |  |  |  |  |
|  |  |  |  |  |  |  |  |  |  |
|  |  |  |  |  |  |  |  |  |  |
|  |  |  |  |  |  |  |  |  |  |
|  |  |  |  |  |  |  |  |  |  |
|  |  |  |  |  |  |  |  |  |  |
|  |  |  |  |  |  |  |  |  |  |
|  |  |  |  |  |  |  |  |  |  |
|  |  |  |  |  |  |  |  |  |  |
|  |  |  |  |  |  |  |  |  |  |
|  |  |  |  |  |  |  |  |  |  |

4.3 By area, education or other factors (Prev. : Prevalence; S/C: standardised or Crude)

| Categories | Age | Number of case | Population | Pre. | S/C |
| --- | --- | --- | --- | --- | --- |
|  |  |  |  |  |  |
|  |  |  |  |  |  |
|  |  |  |  |  |  |
|  |  |  |  |  |  |
|  |  |  |  |  |  |
|  |  |  |  |  |  |
|  |  |  |  |  |  |
|  |  |  |  |  |  |
|  |  |  |  |  |  |
|  |  |  |  |  |  |
|  |  |  |  |  |  |

**S2. Included, excluded and miscellaneous studies**

1. The search strategy of dementia prevalence studies

| **English keywords**  Database: PubMed, Elsevier, PsycInfo, Airti Library | **Chinese keywords**  Database: CNKI and Airti Library |
| --- | --- |
| Publication year: 1980~April 2012 | Publication year: 1980~April 2012 |
|  | Limitation: Medicine and Public Health**^1.^** |
| Prevalence OR epidemiology  AND | Traditional: 患病、流行病、調查、盛行率**^2.^**  Simplified: 患病、流行病、调查 |
|  |  |
| Dementia OR Alzheimer*  AND | Traditional: 老年癡呆、阿爾茨海默  Simplified: 老年痴呆、阿尔茨海默 |
|  |  |
| China OR Chinese OR Taiwan OR Taiwanese | - **^3.^** |

**^1.^**“盛行率”is the translation of “prevalence” in Taiwan while in mainland China, it is translated as “患病率”.

**^2.^** Due to the complex meanings of Chinese keywords, the limitation of subject in medicine and public health was applied in Chinese databases.

**^3.^** The keywords related to location were not applied in Chinese databases.

2. Included studies

| **Id** | **Author** | **Year** | **Location** | **Id** | **Author** | **Year** | **Location** |
| --- | --- | --- | --- | --- | --- | --- | --- |
| **Mainland China** | | | | | | | |
| 1 | Ye G [40] | 2011 | Zheijiang | 48 | Tang M [53] | 1999 | Sichuan |
| 2 | Li L [41] | 2011 | Anhui | 51 | Lou K [54] | 1998 | Shanghai |
| 3 | Zhao Q [42] | 2010 | Shanghai | 53 | Yu J [69] | 1998 | Guangdong |
| 4 | Ji M [43] | 2010 | Shanghai | 54 | Wang J [28] | 1998 | Shandong |
| 5 | Wang F [15] | 2010 | Shandong | 55 | Lv S [55] | 1998 | Zheijiang |
| 7 | Chen B [63] | 2009 | Fujian | 56 | Chen Z [29] | 1998 | Xinjiang |
| 8 | Li H [64] | 2009 | Fujian | 58 | Xue G [70] | 1997 | Guangdong |
| 9 | Wang H [16] | 2009 | Shandong | 59 | Wu C [71] | 1996 | Hainan |
| 10 | Rodriguez [17] | 2008 | 10/66 | 60 | Zhang H [72] | 1996 | Hainan |
| 11 | Wei H [18] | 2008 | Hebei | 62 | Wang D [56] | 1995 | Shanghai |
| 12 | Yan F [19] | 2008 | Beijing | 63 | Wen G [73] | 1995 | Hainan |
| 14 | Huang W [65] | 2007 | Guizhou | 64 | Gao Z [57] | 1994 | Hunan |
| 16* | Tan J [44] | 2007 | Hubei | 65 | Mao R [74] | 1993 | Fujian |
| 18 | Tang M [66] | 2007 | Guangdong | 66 | Gao Z [58] | 1993 | Shanghai, Jiangshu |
| 19 | Zhang Z [79] | 2006 | 4 cities | 69 | Li G [30] | 1989 | Beijing |
| 20 | Zhao D [20] | 2006 | Henan | 70 | Zhang M [59] | 1990 | Shanghai |
| 22* | Tang M [45] | 2005 | Sichuan | 72 | Gao S [31] | 1989 | Beijing |
| 26 | Wang D [67] | 2004 | Guangdong | 73/76 | Chen R [60] | 2011 | Anhui/ 4 Provinces |
| 27 | Tan Z [21] | 2002 | Beijing | 74 | Fan C [32] | 2011 | Shaanxi |
| 28 | Shen C [46] | 2002 | Shanghai | 75 | Kang M [33] | 2011 | Hebei |
| 29 | Zhou K [47] | 2002 | Chongqing | 78 | Chen C [34] | 1992 | Beijing |
| 31 | Zhang Z [22] | 2001 | Beijing | 88 | Yu S [35] | 1994 | Shaanxi |
| 35 | Qu C [23] | 2001 | Shaanxi | 94 | Sun Z [36] | 2012 | Heilongjiang |
| 36 | Sun Y [24] | 2001 | Liaoning | 95 | Pong X [37] | 2010 | Gansu |
| 37 | Fei W [48] | 2000 | Shanghai | 98 | Li K [38] | 2008 | Hebei |
| 38 | Lai S [68] | 2000 | Guangdong | 100 | Yuan Y [61] | 2005 | Jiangshu |
| 39 | Wang W [25] | 2000 | Beijing | 102 | Liang L [75] | 2003 | Guangdong |
| 41 | Zhang Z [49] | 2000 | Shanghai | 103 | Wu C [76] | 2003 | Hainan |
| 42 | Fan J [50] | 2000 | Jiangshu | 109 | Tang X [39] | 1998 | Beijing |
| 43 | Li S [26] | 1999 | Beijing | 111 | Chen W [77] | 2004 | Hainan |
| 44 | Li Z [27] | 1999 | Beijing | 146 | Cheng Z [62] | 1989 | Shanghai |
| 46 | Xiao Z [51] | 1999 | Hunan | 149 | Lai M [78] | 2011 | Hainan |
| 47 | Wang T [52] | 1999 | Anhui |  |  |  |  |
| **Hong Kong** | | | | | | | |
| 50 | Chiu H [80] | 1998 | Hong Kong |  |  |  |  |
| **Taiwan** | | | | | | | |
| 52 | Lin R [81] | 1998 | KaoKaoPing | 84 | Yip P [84] | 1992 | Taipei |
| 57 | Liu H [82] | 1998 | Kinmen | 85 | Lee C [85] | 1997 | Ilan |
| 61 | Liu C [83] | 1996 | Kaohsiung | 159 | Liu H [86] | 1995 | Taiwan |

*These two studies reported the same data.

3. Excluded studies

| **Id** | **Author** | **Year** | **Location** | **Excluded reason** |
| --- | --- | --- | --- | --- |
| **Duplicate publication** | | | | |
| 15 | Li S | 2007 | Beijing | Duplicate with 12 (Yan et al., 2008) |
| 21 | Zhang Z | 2005 | 4 Cities | Duplicate with 19 (Zhang et al., 2006) |
| 23 | Ma S | 2005 | Guangdong | Duplicate with 18 (Tang et al., 2007) |
| 32 | Zhou F | 2001 | Shanghai | Duplicate with 3 (Zhao et al., 2010) |
| 33 | Zhang G | 2001 | Shanghai | Duplicate with 41 (Zhang et al., 2000) |
| 34 | Tang M | 2001 | Sichuan | Duplicate with 22 (Tang et al., 2005) |
| 45 | Gao Z | 1999 | Shanghai | Duplicate with 66 (Gao et al., 1993) |
| 68 | Zhang M | 1990 | Shanghai | Duplicate with 70 (Zhang et al., 1990) |
| 82 | Shen Y | 1994 | Shanghai | Duplicate with 78 (Chen et al., 1992) |
| 107 | Sun C | 1999 | Anhui | Duplicate with 47 (Xiao et al., 1999) |
| 110 | Sun Z | 2011 | Heilongjiang | Duplicate with 94 (Sun et al., 2012) |
| 119 | Chen S | 1996 | Xinjiang | Duplicate with 56 (Chen et al., 1998) |
| 123 | Pong X | 2009 | Gansu | Duplicate with 95 (Gao et al., 2009) |
| 133 | Li S | 1994 | Beijing | Duplicate with 69 (Li et al., 1989) |
| 141 | Hong Z | 2000 | Shanghai | Duplicate with 3 (Zhao et al., 2010) |
| 160 | Shen Y | 1994 | Beijing | Duplicate with 69 (Li et al., 1989) |
| 161 | Liu H | 1997 | Taiwan | Duplicate with 57 (Liu et al., 1998) |
| 162 | Shen Y | 1994 | Beijing | Duplicate with 69 (Li et al., 1989) |
| **Follow-up studies** | | | | |
| 49 | Zhu Z | 1998 | Shanghai | Follow-up study of fixed population |
| 99 | Qu C | 2005 | Shaanxi | Follow-up study of fixed population |
| 104 | Yan F | 2001 | Beijing | Follow-up study of fixed population |
| 138 | Tang Z | 2003 | Beijing | Follow-up study of fixed population |
| **Studies which violated inclusion criteria^1.^** | | | | |
| 79 | Zhang C | 1998 | Beijing | Violated inclusion criteria (1) |
| 81 | Young C | 2003 | Xinjiang | Violated inclusion criteria (1) |
| 96 | Gao Y | 2009 | Shanxi | Violated inclusion criteria (1) |
| 112 | Po C | 1992 | Tianjin | Violated inclusion criteria (1) |
| 134 | Xie C | 2003 | Shandong | Violated inclusion criteria (1) |
| 154 | Kuo M | 2010 | Shaanxi | Violated inclusion criteria (1) |
| 105 | Zhu C | 2000 | Shandong | Violated inclusion criteria (1) (2) |
| 142 | Wei S | 1996 | Xinjiang | Violated inclusion criteria (1) (4) |
| 155 | Zhang C | 2009 | Shanxi | Violated inclusion criteria (1) (4) |
| 114 | Tang W | 2005 | Zhejiang | Violated inclusion criteria (3) |
| 115 | Chen H | 2004 | Jiangxi | Violated inclusion criteria (3) |
| 116 | Zhang C | 2003 | Zhejiang | Violated inclusion criteria (3) |
| 117 | Zhang C | 2003 | Zhejiang | Violated inclusion criteria (3) |
| 118 | Huang C | 2001 | Guangdong | Violated inclusion criteria (3) |
| 121 | Yu C | 2009 | Zhejiang | Violated inclusion criteria (3) |
| 125 | Liang Z | 2010 | Guangxi | Violated inclusion criteria (3) |
| 127 | Wang X | 2008 | Zhejiang | Violated inclusion criteria (3) |
| 131 | Zhu H | 2004 | Jiangxi | Violated inclusion criteria (3) |
| 137 | Zhang W | 1998 | 7 regions | Violated inclusion criteria (3) |
| 140 | Wang S | 2005 | Zhejiang | Violated inclusion criteria (3) |
| 143 | Ye T | 1995 | Hainan | Violated inclusion criteria (3) |
| 153 | Wu H | 2003 | Jiangxi | Violated inclusion criteria (3) |
| 113 | Qu K | 1991 | Shanghai | Violated inclusion criteria (3) (4) |
| 144 | Qu K | 1989 | Shanghai | Violated inclusion criteria (3) (4) |
| 150 | Zhou K | 2011 | Jiangsu | Violated inclusion criteria (3) (4) |
| 152 | Chen Y | 2004 | Zhejiang | Violated inclusion criteria (3) (4) |
| 25 | Wei L | 2004 | Inner Mongo | Violated inclusion criteria (4) |
| 30 | Sun C | 2002 | Yunnan | Violated inclusion criteria (4) |
| 40 | Wei M | 2000 | Beijing | Violated inclusion criteria (4) |
| 67 | Song S | 1993 | Shandong | Violated inclusion criteria (4) |
| 80 | Weng L | 1995 | Hubei | Violated inclusion criteria (4) |
| 86 | Rin H | 1987 | Taiwan | Violated inclusion criteria (4) |
| 89 | Zhao YZ | 1986 | 12 regions | Violated inclusion criteria (4) |
| 93 | Zhao M | 1991 | Guizhou | Violated inclusion criteria (4) |
| 97 | Pong I | 2008 | Hunan | Violated inclusion criteria (4) |
| 128 | Cheng Y | 2007 | Guangdong | Violated inclusion criteria (4) |
| 129 | Liang W | 2005 | Guangdong | Violated inclusion criteria (4) |
| 132 | Rencindaoqi | 2004 | Inner Mongo | Violated inclusion criteria (4) |
| 139 | Huo D | 2000 | Shaanxi | Violated inclusion criteria (4) |
| 145 | OuYang S | 1989 | Sichuan | Violated inclusion criteria (4) |
| 148 | Gao Z | 1988 | Shanghai | Violated inclusion criteria (4) |
| 156 | Shen Y | 2000 | 7 regions | Violated inclusion criteria (4) |
| **The studies with other main focus** | | | | |
| 6 | Sousa | 2010 | 10/66 | Focused on the dependency of elderly |
| 13 | Lam | 2008 | Hong Kong | Focused on mild dementia |
| 17 | Wang S | 2007 | Liaoning | Focused on BPSD |
| 71 | Tsai | 1989 | Shanghai | Focused on validity of HDS |
| 101 | Xie H | 2004 | Beijing | Focused on psychological and neurological symptom |
| 106 | Tang M | 1999 | Sichuan | Focused on MCI and dementia |
| 108 | Pong H | 1998 | Shanghai | Focused on MMSE cut-offs |
| 122 | Xiang J | 2009 | Hubei | Focused on MMSE cut-offs |
| 124 | Yao Y | 2009 | Shanghai | Focused on MMSE cut-offs |
| 130 | Li H | 2005 | Guangdong | Focused on General health of older people |
| 77 | Wang Z | 1995 | Guangdong | Focused on Alzheimer’s disease only |
| 157 | Huriletermuer | 2011 | Inner Mongolia | Focused on Alzheimer’s disease only |
| 24 | Shen C | 2004 | Shanghai | Incidence |
| 90 | Li G | 1989 |  | Overview |
| 158 | Liu H | 1994 | Taiwan | Pilot study of Liu et al., 1998 with resampling population |

**^1.^** Inclusion criteria: (1) The study was a field survey; (2) The study included a population sampling (3) The study reported the prevalence in the population aged 50 and over (4) Dementia case was not decided by one screening test and diagnosis criteria were reported in the study.

| 4. Miscellaneous studies | | | | | | | | | |
| --- | --- | --- | --- | --- | --- | --- | --- | --- | --- |
| **Study** | **Location** | **N** | **Resp. rate** | **Type** | **Age** | **Screening tools** | **Diagnostic criteria/ Instrument** | **Results** | **Note** |
| Lin et al., 1982 | Taiwan | 1057 | - | Rural | 65+ | HDS | Clinical diagnosis, interview by psychiatrists | 2.7%  Men:1.0%; Women:4.5% | Subjective diagnosis |
| Kuang et al., 1984 | Hubei | 7340 | - | Urban | 60+ | - | Diagnosis criteria based on Chinese Society of Psychiatry, 1987 | 0.068% | General mental disorders |
| Chen et al., 1987 | Beijing | 8740 | - | Urban | 60+ | PSE for general mental disorders | Clinical diagnosis based on the Handbook of Mental Disorders and Epidemiological Investigation, CT | 0.49% | General mental disorders |
| Kuang et al., 1993 | Jiangxi | 2380 | - | Mixed | 60+ | HDS (<15) | Clinical diagnosis by psychiatrists | 3.68% | Subjective diagnosis |
| Hou et al., 1996 | Jiangsu | 470 | - | Mixed | 6+ | PSE for general mental disorders | Clinical diagnosis based on the Handbook of Mental Disorders and Epidemiological Investigation | 60+: 0.21% | General mental disorders |
| Cong et al.,  2008 | Hebei | 9021 | 89.6% | Mixed | 18+ | MMSE | DSM-IV | 50-59: 1.13%  60-69: 4.46%  70-79:12.81% | Early and late onset of dementia |
| Qu et al., 2003 | Shanghai | 1325 | 85.7% | Urban | 65+ | CRBRS, PSE | Clinical diagnosis based on the Handbook of Mental Disorders and Epidemiological Investigation | 0.453 | General mental disorders |
| Liu et al.,  1998 | 7 regions | 3341 | - | Mixed | 60+ | 10-question screening PSE, mental status scale, SDSS | ICD-10, CCMD-2, the criteria of Chinese Society of Psychiatry, 1987 | AD: 0.21% | General mental disorders |
| Deng et al., 1988 | Sichuan | 1219 | - | Mixed | 60+ | MMSE, CES-D, mental status scale | Clinical diagnosis based on the Handbook of Mental Disorders and Epidemiological Investigation | 0.66% | General mental disorders |
| Zhang et al.,  2004 | Anhui | 11940 | 87.4% | Mixed | 15+ | PSE for general mental disorders | Clinical diagnosis based on the Handbook of Mental Disorders and Epidemiological Investigation | AD, 60+: 0.92%  AD, 65+: 1.24% | General mental disorders |

**Abbreviations**: AD: Alzheimer’s disease; CCMD-2: Chinese Classification of Mental Disorders, 2; CES-D: Centre for Epidemiology Studies Depression Scale; CRBRS: Crichton Royal Behaviour Rating Scale; DSM-IV: Diagnosis and Statistical Manual of Mental Disorders, IV; HDS: Hasegawa Dementia Scale; MMSE: Mini Mental Status Examination; PSE: Present State Examination; SDSS: Social Disability Screening Scale


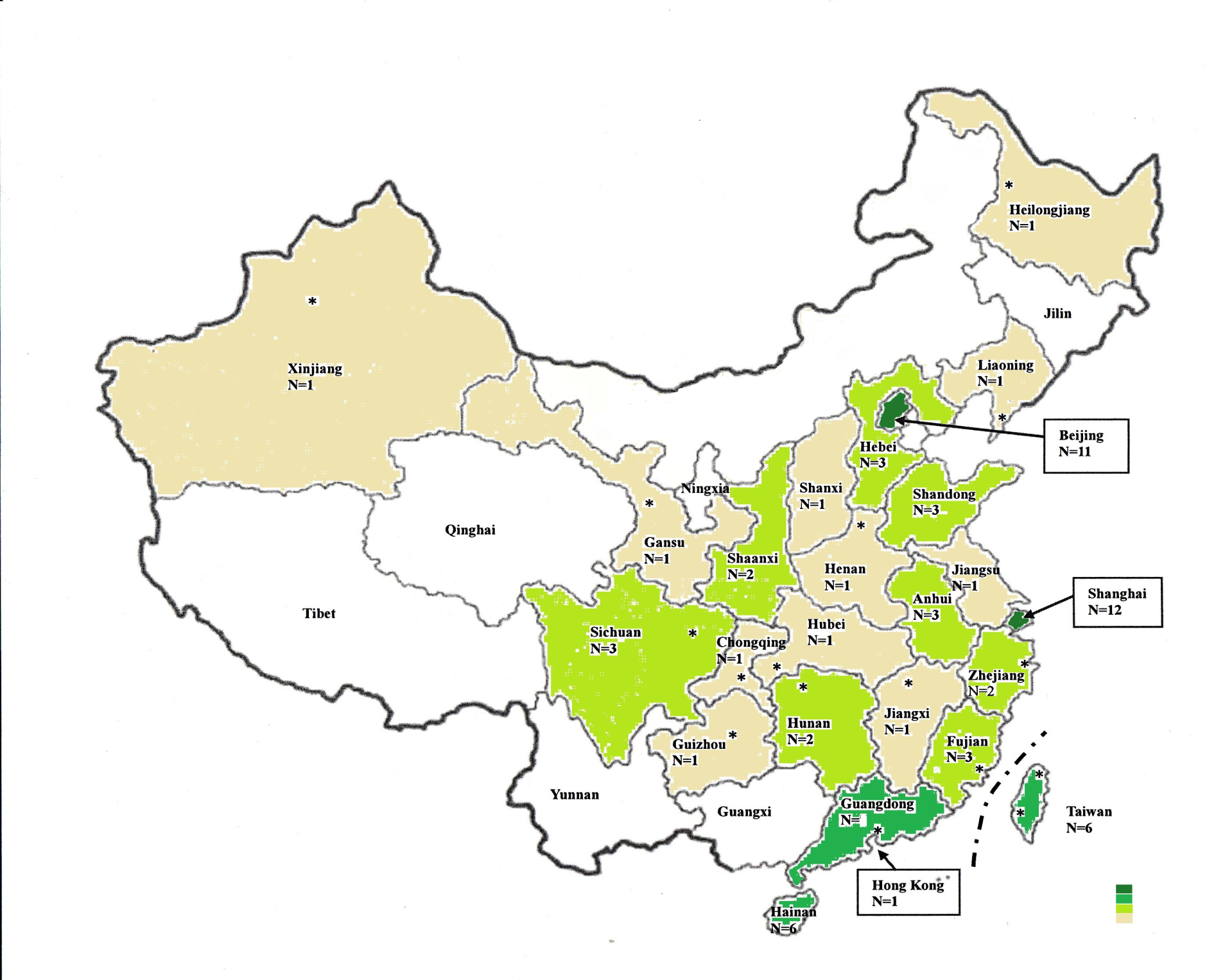


**6**

**11+**

**6-10**

**2-5**

**1**

**Number of studies**

**Inner Mongolia**

**Nationwide studies**

**4 cities:**

Beijing,

Shanghai,

Chengdu,

Xian

**4 provinces:** Shanghai,

Shanxi,

Guangdong,

Heilongjiang

**Figure S1: Included studies by provinces**

**S3. Estimating the number of people with dementia in mainland China, Hong Kong and Taiwan**

**1. Introduction**

The scientific evidences derived from the existing prevalence studies of dementia in mainland China, Hong Kong and Taiwan can provide important message for public health implication. The aim of estimating the numbers of people with dementia in nationwide and different areas is to apply the findings in the systematic review and meta-analysis to generate the detailed information of prevalence and estimate the population with dementia in mainland China, Hong Kong and Taiwan.

Based on the results of meta-analysis, the significant associations between various methodological factors (diagnostic criteria, whole study age range, study size and sampling method), geographical variations and the prevalence of dementia were found. The pooled estimates of crude prevalence by age groups approximately followed the pattern of doubling every 5 years. In order to estimate the number of population with dementia and consider the effect of methodological factors and regional differences, more detailed estimate of prevalence by 5-year age groups was generated from the results in meta-regression model and applied to national and regional demographics to estimate the number of people with dementia in different areas.

**2. The estimation of age-stratified prevalence**

**2.1 Method**

The calculation method of age-stratified prevalence by 5-year age groups is described as below. Based on the findings in the meta-analysis, it was assumed that:

(1) P= prevalence of dementia in people aged 60 and over; the overall estimates in meta-regression models

(2) $p_{1=}$prevalence of age 60-64

$p_{2=}$ prevalence of age 65-69

$p_{3=}$ prevalence of age 70-74

$p_{4=}$ prevalence of age 75-79

$p_{5=}$ prevalence of age 80-84

$p_{6=}$ prevalence of age 85-89

$p_{7=}$ prevalence of age 90 and over

(3) Based on the results of crude prevalence by age groups, the prevalence approximately double every 5 years:

$p_{2=}$2$p_{1}$_;_ $p_{3=}$4$p_{1}$_;_ $p_{4=}$8$p_{1}$_;_ $p_{5=}$16$p_{1}$_;_ $p_{6=}$32$p_{1}$_;_ $p_{7=}$64$p_{1}$

Then, the age-stratified prevalence of dementia can be calculated as

$p_{1=}\frac{P}{\sum_{i=1}^{7} 2^{(i-1)}\times r_{i}}$, $r_{i}$= the proportion of the age group in the study population

According to (3), the $p_{2\sim7}$ can be calculated by $p_{1}$

Based on the result of meta-analysis, the ratio of overall prevalence in women to men is about 1.7 and the prevalence by age and gender in different diagnostic criteria were estimated by adjusting this ratio.

Since diagnostics criteria importantly affected the results and varied largely in the existing studies, the numbers of people with dementia were estimated and demonstrated separately by two main types of diagnostic criteria, DSM-III and DSM-IV, which considerably influenced the identification of dementia cases.

**2.2 Results**

The results of the estimated prevalence by different diagnostic criteria were compared with the overall estimates of crude prevalence and demonstrated in Figure S2. The estimated prevalence was similar to the crude prevalence. The estimated prevalence based on DSM-IV was nearly twice higher than DSM-III across all age groups. Nearly half of DSM-IV and the estimates of all types of diagnostic criteria were the median of DSM-III and DSM-IV.

Table S1 presented the age-stratified prevalence by diagnostic criteria and four different areas, including North, Central, South China and Hong Kong/ Taiwan. The estimates in meta-regression model indicated about 3% lower prevalence of people aged 60 and over in Hong Kong and Taiwan compared to North China. Most of the studies in Hong Kong and Taiwan included their participants from 65 years old. Only one study in Taiwan investigated people aged 60 and over (Liu et al., 1995). The study reported especially low prevalence (1.6%, 95% CI: 1.1, 2.2) and importantly caused the low estimated prevalence in the meta-regression model. After removing the only study with the age range 60 and above, the estimate reduced to zero, which is an unreasonable number and purely caused by modelling. The estimated prevalence of the age range 65 and over in the regression model is considered to be more reliable and appropriate to use this estimate to generate 5-year age-stratified prevalence from 65 in Hong Kong and Taiwan.

**Figure S2:** The comparison of crude and inference prevalence

**Table S1:** Estimated prevalence of 5-year age groups by diagnostic criteria and different areas

|  | **Total ^a.^** | **60-64** | **65-69** | **70-74** | **75-79** | **80-84** | **85-89** | **90+** |
| --- | --- | --- | --- | --- | --- | --- | --- | --- |
| DSM-III Pre_60, North | 3.8 (2.6, 4.9) | 0.7 | 1.4 | 2.7 | 5.4 | 10.8 | 21.7 | 43.3 |
| DSM-III Pre_60, Central | 2.6 (1.4, 3.8) | 0.5 | 0.9 | 1.9 | 3.8 | 7.5 | 15.1 | 30.2 |
| DSM-III Pre_60, South | 1.9 (0.5, 3.3) | 0.3 | 0.7 | 1.3 | 2.7 | 5.4 | 10.7 | 21.5 |
| DSM-III Pre_60, HK/ TW | 0.4 (0.0, 2.4) | 0.1 | 0.2 | 0.3 | 0.6 | 1.2 | 2.4 | 4.7 |
| DSM-III Pre_65, HK/ TW | 2.8 (0.8, 4.8) |  | 0.7 | 1.4 | 2.9 | 5.7 | 11.5 | 23.0 |
|  |  |  |  |  |  |  |  |  |
| DSM-IV Pre_60, North | 5.4 (4.3, 6.5) | 1.0 | 2.0 | 3.9 | 7.9 | 15.7 | 31.4 | 62.9 |
| DSM-IV Pre_60, Central | 4.3 (3.1, 5.5) | 0.8 | 1.6 | 3.1 | 6.2 | 12.4 | 24.8 | 49.7 |
| DSM-IV Pre_60, South | 3.6 (2.1, 4.9) | 0.6 | 1.3 | 2.6 | 5.1 | 10.3 | 20.5 | 41.0 |
| DSM-IV Pre_60, HK/ TW | 2.1 (0.2, 4.1) | 0.4 | 0.8 | 1.5 | 3.0 | 6.1 | 12.1 | 24.3 |
| DSM-IV Pre_65, HK/TW | 4.5 (2.5, 6.5) |  | 1.2 | 2.3 | 4.6 | 9.2 | 18.4 | 36.9 |

^a.^ Based on the results of meta-analysis; pooled prevalence with the adjustment of methodological factors and 95% CI

**3. Estimated number of people with dementia**

**3.1 Method**

The age-stratified prevalence in the previous section was applied to the population in mainland China, Hong Kong and Taiwan to estimate the number of people with dementia. The sum of population estimates were obtained from the sixth Nation Population Census in 2010 by National Bureau of Statistics of China, official statistics of Department of Interior, Taiwan and Census and Statistics Department, Hong Kong Special Administrative regions (SAR). The extracted data included the overall population, the numbers of population by men and women, by province and cities.

Similar method was used to estimate the numbers of people with dementia in next 50 years in mainland China, Hong Kong and Taiwan. The change of population structures in mainland China were obtained from World Population Prospects, the 2010 revision and used estimate the pattern of the people with dementia (United Nations, 2011). The population structures of Hong Kong and Taiwan in next 50 years were obtained from official statistics of Department of Interior, Taiwan and Census and Statistics Department, Hong Kong Special Administrative regions (SAR).

**3.2 Results**

The age-stratified prevalence was applied to the population in mainland China, Hong Kong and Taiwan. The estimated numbers of people with dementia by areas, provinces and cities are provided in Table S2. The estimated numbers based on the newer standard of diagnosis, DSM-IV, are demonstrated in the main text of the paper.

The estimated numbers of people with dementia in Hong Kong and Taiwan was mainly focused on age groups over 65. The 5-year age-stratified prevalence from 65 years old was applied to estimate the number of people with dementia and the prevalence was expected to be higher than the prevalence in mainland China which was estimated from age 60 and above.

The provinces in “West China”, such as Xinjiang, Tibet, Inner Mongo, Qinghai and Ningxia, are less developed provinces with high proportion of ethnic minority. It is expected that the age structure and living conditions in these provinces are different from the provinces in east area. However, there are few studies which can be used to generate reliable estimates. In the previous analysis, these provinces were categorised into north China for approximate estimates since the two studies in Xinjiang and Gansu provinces were in urban area, which are expected to be similar to cities in North China. The model of north area might over-estimate the number of people with dementia in these provinces. Especially many studies in northern area were conducted in Beijing.

The prevalence in Table S2 slightly varies from the estimates in meta-regression model. This might attribute to the different age structures of the population in various provinces and areas. The prevalence in North China is lower than the estimate in the meta-regression model. This might be importantly related to the younger age structure in the western area. The prevalence of Hong Kong and Taiwan in Table S2 is higher than the estimate, which summarised the prevalence studies in 1990s by meta-regression. The population ageing in Hong Kong and Taiwan was expected to increase the prevalence of dementia.

The results of projected number of people with dementia in next 50 years are demonstrated in Table S3. The numbers of people with dementia in the three places were expected to raise considerably and double in every 20 years. Especially in mainland China, the population with dementia is estimated to exceed 20 million by 2030 and approach 50 million by 2060.

**Table S2:** Estimated numbers of people with dementia by areas, provinces and cities

|  | **Older population**  (60+ in mainland China; 65+ in Hong Kong and Taiwan, million) | **DSM-IV**  (million) | **Prevalence**  (%) | **DSM-III**  (million) | **Prevalence**  (%) |
| --- | --- | --- | --- | --- | --- |
| **Mainland China**  (age 60+) | **177.6** | **8.18** | **4.61** | **5.17** | **2.91** |
| **Hong Kong**  (age 65+) | **0.9** | **0.06** | **6.80** | **0.05** | **5.56** |
| **Taiwan**  (age 65+) | **2.5** | **0.15** | **5.70** | **0.09** | **3.60** |
| **North China** | **73.37** | **3.76** | **5.13** | **2.59** | **3.53** |
| Beijing | 2.46 | 0.14 | 5.69 | 0.10 | 4.06 |
| Hebei | 9.34 | 0.46 | 4.92 | 0.32 | 3.43 |
| Shanxi | 4.12 | 0.20 | 4.86 | 0.14 | 3.40 |
| Liaoning | 6.75 | 0.36 | 5.33 | 0.25 | 3.70 |
| Heilongjiang | 4.99 | 0.23 | 4.61 | 0.16 | 3.20 |
| Shandong | 14.13 | 0.81 | 5.73 | 0.56 | 3.96 |
| Henan | 11.97 | 0.64 | 5.35 | 0.44 | 3.68 |
| Shaanxi | 4.80 | 0.22 | 4.59 | 0.15 | 3.13 |
| Gansu^2^ | 3.18 | 0.13 | 4.09 | 0.09 | 2.83 |
| Xinjiang^2^ | 2.11 | 0.10 | 4.74 | 0.07 | 3.32 |
| Jilin^1^ | 3.63 | 0.17 | 4.69 | 0.12 | 3.31 |
| Inner Mongo^1,2^ | 2.84 | 0.13 | 4.58 | 0.09 | 3.17 |
| Tianjin^1^ | 1.68 | 0.09 | 5.34 | 0.06 | 3.56 |
| Tibet^1,2^ | 0.23 | 0.01 | 4.34 | 0.01 | 4.34 |
| Qinghai^1,2^ | 0.53 | 0.02 | 3.76 | 0.02 | 3.76 |
| Ningxia^1,2^ | 0.61 | 0.03 | 4.92 | 0.02 | 3.28 |
| **Central China** | **73.30** | **3.22** | **4.39** | **1.95** | **2.66** |
| Shanghai | 3.47 | 0.19 | 5.48 | 0.11 | 3.17 |
| Jiangsu | 12.57 | 0.58 | 4.61 | 0.35 | 2.78 |
| Zhejiang | 7.56 | 0.36 | 4.76 | 0.22 | 2.91 |
| Anhui | 8.93 | 0.39 | 4.37 | 0.23 | 2.58 |
| Jiangxi | 5.10 | 0.22 | 4.31 | 0.13 | 2.55 |
| Hubei | 7.97 | 0.31 | 3.89 | 0.19 | 2.38 |
| Hunan | 9.56 | 0.41 | 4.29 | 0.25 | 2.62 |
| Chongqing | 5.02 | 0.21 | 4.18 | 0.13 | 2.59 |
| Sichuan | 13.11 | 0.55 | 4.20 | 0.33 | 2.52 |
| **South China** | **30.93** | **1.20** | **3.88** | **0.63** | **2.04** |
| Fujian | 4.21 | 0.17 | 4.04 | 0.09 | 2.14 |
| Guangdong | 10.15 | 0.43 | 4.24 | 0.23 | 2.27 |
| Hainan | 0.98 | 0.04 | 4.07 | 0.02 | 2.04 |
| Guangxi^1^ | 6.04 | 0.24 | 3.98 | 0.13 | 2.15 |
| Guizhou^1^ | 4.46 | 0.14 | 3.14 | 0.07 | 1.57 |
| Yunnan^1^ | 5.09 | 0.18 | 3.54 | 0.09 | 1.77 |

^1.^ Provinces with no studies to date ^2.^ For the border provinces in western areas with few existing studies, prevalence was approximately estimated modelled on prevalnce in other areas of the north.

**Table S3:** Projected number of people with dementia from 2012 to 2060 in mainland China, Hong Kong and Taiwan (unit: million)

| **DSM-IV** | **Current** | **2020** | **2030** | **2040** | **2050** | **2060** |
| --- | --- | --- | --- | --- | --- | --- |
| Mainland China (age 60+) | 8.18 | 13.5 | 20.3 | 29.59 | 40.63 | 48.68 |
| Hong Kong (age 65+) | 0.06 | 0.09 | 0.13 | 0.20 | 0.29 | 0.32 |
| Taiwan (age 65+) | 0.15 | 0.21 | 0.32 | 0.49 | 0.66 | 0.71 |
| **DSM-III** |  |  |  |  |  |  |
| Mainland China (age 60+) | 5.17 | 6.86 | 10.32 | 15.05 | 20.66 | 24.75 |
| Hong Kong (age 65+) | 0.05 | 0.06 | 0.08 | 0.13 | 0.18 | 0.20 |
| Taiwan (age 65+) | 0.09 | 0.13 | 0.20 | 0.31 | 0.41 | 0.44 |
